# Catalytic evaluation of cellulose pyrolysis by using nanosized metal oxide catalysts

**DOI:** 10.55730/1300-0527.3522

**Published:** 2022-10-08

**Authors:** Yusuf Osman DONAR, Ali Tolga ÜNALAN, Samed ERGENEKON, Ali SINAĞ

**Affiliations:** Department of Chemistry, Faculty of Science, Ankara University, Ankara, Turkey

**Keywords:** Titanium dioxide, zinc oxide, cellulose, pyrolysis, catalyst

## Abstract

In this study, effects of TiO_2_ and ZnO nanometal oxides on cellulose pyrolysis have been investigated. Both catalysts have been synthesized via hydrothermal method and characterized by using different techniques. Catalytic and catalyst-free experiments were carried out so as to identify the catalytic abilities of synthesized nanoparticles. Catalyst-free experiments were carried out at 500, 600, and 700 °C in order to determine the optimal condition for pyrolysis and it was found as 700 °C. Optimum catalyst ratio for cellulose pyrolysis was found as 5% (w/w) for both TiO_2_ and ZnO catalysts. GC-MS and micro-GC analyses were conducted in order to examine the catalytic properties of synthesized nanoparticles and illuminate the content of pyrolytic oil and gaseous products. Results showed that maximum gas yield was observed at 700 °C in the presence of 5% TiO_2_. Maximum activity for both catalysts was observed at 700 °C and the char yield was significantly decreased in each catalytic experiment at specified temperatures, compared to catalyst-free experiments. Both nanoparticles catalyzed the dehydration and decarbonylation reactions and significantly increased the amount of furan derivatives, especially furanic aldehydes.

## 1. Introduction

Metal oxides have gained great importance over the last years thanks to their remarkable properties such as nanocrystal shape, polar surface, and acidity/alkalinity [1–4]. In catalytic pyrolysis of biomass, metal oxides are not widely used and their effects are not fully elucidated [5]. The identification of the catalytic performance of nanosized metal oxides on cellulose pyrolysis is important in terms of developing a renewable energy source and improving its features. Various studies have been conducted for the beneficial use of cellulose such as gasification and high-temperature pyrolysis but both techniques have poor selectivity and need more energy to be conducted [6–8]. Enzyme-catalyzed cellulose hydrolysis has been studied but the enzymatic route is slow and the entire process is quite costly [8–10].

Scientists have studied various catalysts for upgrading pyrolysis vapors [10]. In Lu’s study, catalytic effects of sulfated titania on cellulose pyrolysis have been investigated. As a result, sulfated titania improved selectivity [11]. In another study, effects of ZrO2/TiO2 and ZSM-5 catalysts have been investigated on guaiacol, cellulose, and wood pyrolysis. As a result, both catalysts are potential candidates to improve selectivity [12]. Rutile and anatase TiO_2_ (commercial) catalysts and their modified ones with incorporation of Ce, Ru, and Pd were used on pyrolysis of poplar wood [13]. By using the solvothermal method, TiO_2_ nanoparticles were synthesized and tested on low-rank coal pyrolysis. The results indicated an increase in gaseous product yields [14].

Several reports have been published on the investigation of catalytic effect of zinc oxide on biomass pyrolysis. Nokkosmaki et al. [15] have studied the conversion of pyrolytic vapor of pine sawdust, in the presence of ZnO nanoparticles and they emphasized on the significant decrease of tar product yield. ZnO nanoparticles did not show any effect on organic phase but decomposed the anhydrous sugars and polysaccharides present in water phase. Another important output was that the product interacted with ZnO was obtained with a lower viscosity, compared to the raw product [15]. The effect of ZnO nanoparticles on the conversion of cellulose was investigated and the results showed that ZnO nanoparticles led to improve hydrogen yield [16]. In order to bring further clarification on this matter, TiO_2_ and ZnO catalysts have been synthesized and used on cellulose pyrolysis. Effects of synthesized catalysts on product distribution at different temperatures were investigated.

For the differentiation of the catalytic effect, pyrolysis experiments were conducted with and without catalyst. In order to observe the effects of catalysts on pyrolysis experiments, the products obtained from the pyrolysis experiments were compared to each other and characterized by GC/MS and micro-GC analyses.

## 2. Experimental

### 2.1. Materials and methods

For the synthesis of TiO_2_ nanoparticles_,_ 3 mL of titanium tetraisopropoxide (TTIP) was used as Ti source. Presence of alkoxide groups that are attached to titanium leads to an advantageous method for obtaining metal oxides from metal alkoxides since they have relatively large molecular mass and are easily separated groups. 2-propanol was placed in autoclave as a solvent and 3 mL of TTIP were added. One milliliter of distilled water were added dropwise and under vigorous stirring for 15 min at 4000 rpm. After 15 min, white Ti(OH)_4_ was obtained in the solution. Afterwards, the autoclave was sealed and stirred at 4000 rpm speed for 6 h at 140 °C. The reaction was carried out in these conditions. Maximum autogenous pressure throughout the experiment was measured as 10 bar.

Autoclave was cooled after the experimental period ended. Thenceforward, to obtain the particles, the solution was centrifuged and washed with 50 mL of ethyl alcohol and then washed again with 50 mL of distilled water to remove any possible impurities. The particles were left to be dried at 75 °C for 6 h.

For the synthesis of ZnO nanoparticles, 30 mL of ethyl alcohol, 2.5 g of Zn(OAc)_2_.2H_2_O, and 0.5 g of CTAB as a surfactant were added in autoclave and stirred at 4000 rpm speed for 15 min. At the end of the process, autoclave was closed. The reaction was performed at 140 °C for 6 h and the maximum autogenous pressure throughout the reaction was measured as 4 bar.

The autoclave was left to be cooled at room temperature after the experimental period ended. The particles present in the solution were obtained by centrifuge and washed with 100 mL of hot water and 50 mL of ethyl alcohol. At the end of the process, particles were left to be dried at 75 °C for 6 h.

### 2.2 Experimental set-up of pyrolysis system

**Figure 1:**
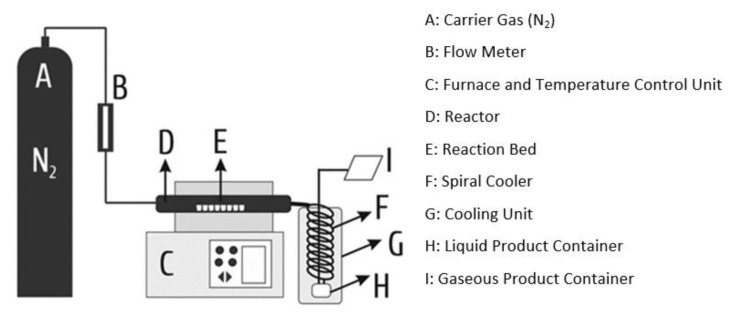
Experimental setup of pyrolysis system.

Pyrolysis was conducted in a quartz reactor containing a tubular furnace. In catalyst-free experiments, 3 g of cellulose sample was placed into a quartz boat. In catalytic experiments, specified amount of cellulose and catalyst were mixed and placed inside of the reactor. Subsequently, the outlet of the quartz reactor was connected to the cooling unit and the cooling unit connected to the gas collection unit (tedlar bag). Spiral cooling system was placed into the Dewar container. Dewar container was filled with ice-ethyl alcohol mixture in order to keep the temperature in the range of −8 ± 2 °C. Prevacuumed gas collecting container was used as a gas-collecting unit. Thus, the liquid product was collected by condensing the gas-vapor mixture coming from the reactor on a spiral glass that cooled with ethyl alcohol-ice mixture and the gaseous product was passed to the collecting container. In order to provide an inert atmosphere, the system was purged with N_2_ gas for half an hour with the flow rate of 30 mL min^−1^ before the experiment. After the purging process, the system was disconnected entirely from the external atmosphere; thus, an inert atmosphere was provided throughout the experimental period. The pyrolysis system used in this study is shown in Figure 1. Pyrolysis of cellulose was carried out at desired temperatures (500, 600, and 700 °C) for an hour. For all experiments, heating rate was set at 50 °C min^−1^. Fifteen minutes after the end of the experiment, the system was purged with nitrogen gas with 30 mL min^−1^ flow speed for 15 min to collect the remaining gaseous products in the system.

Optimum temperature for total conversion (liquid yield + gas yield) was determined by using the results of catalyst-free experiments. The effect of catalyst ratio was observed at constant temperature with ratios of 1%, 5%, and 15%. Optimum catalyst ratio was determined and this ratio was applied at 500 °C, 600 °C, and 700 °C. All experiments were repeated three times and optimum results obtained from these repeated experiments were collected and used.

### 2.3 Analysis of gaseous and liquid products

The analyses of the gaseous products were conducted via micro-GC (SRA instruments T-3000). This instrument consists of two modules (MS5A and PPU) with TCD detector. Argon and helium were used as carrier gases. These modules provide detailed identification for the products. The output from the instrument was % mole and the equation used to convert % mole to mg gas/g biomass is given in Equations (1–3) [17].


(1) Total gas amount (g)=[initial mass of cellulose (g)×gas yield (%)]/100

A: Total mole number of gaseous products (mole)


(2) $$A=\frac{total\;gas\;amount\;(g)}{\langle M\rangle \;of\;gaseous\;products\;(g.{mol}^{-1})}$$

(3) $$\frac{mg\;gas}{g\;cellulose}=\frac{A\times mold\;\%\;of\;the\;gas}{100}\times \frac{molar\;mass\;of\;the\;gas}{initial\;mass\;of\;cellulose}$$

GC-MS analyses were conducted by using Shimadzu GC-MS-QP2010 PLUS. Applied procedure was given in detail elsewhere [17]. The amount of liquid product has been obtained by weight difference between spiral cooler (F in Figure 1) and liquid product container (H in Figure 1), before and after the experiments. Gas percentage was obtained by subtracting the solid and liquid product amounts from the mass of pyrolyzed cellulose.

## 3. Results and discussion

### 3.1. Material characterization

For the characterization of the nanoparticles; X-Ray Diffraction (XRD), transmittance electron microscope (TEM), Brunauer–Emmett–Teller (BET), and temperature programmed desorption (NH_3_-TPD) analyses were conducted. Gas chromatography mass spectrometry (GC-MS) and micro-GC analyses were performed in order to characterize the liquid and gaseous products.

**Figure 2:**
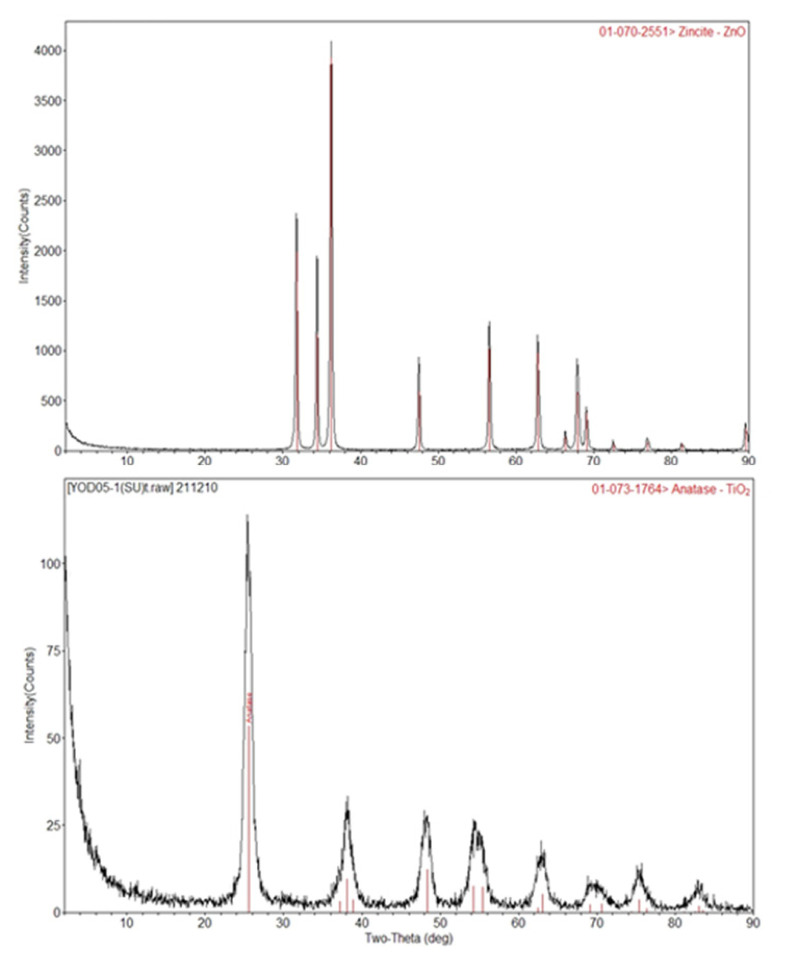
XRD patterns of ZnO (above) and TiO_2_ (below).

XRD patterns of synthesized nanoparticles were analyzed by using Rigaku D/Max-2200 ULTIMAN X-ray diffractometer using Cu-Kα radiation (λ = 1.5418). For the interpretation of obtained patterns; Joint Committee on Powder Diffraction Standards (JCPDS), Powder Diffraction File (PDF), and International Centre for Diffraction Data (JCDD) files were used. XRD patterns of ZnO and TiO_2_ nanoparticles are shown in Figure. 2. According to XRD results of ZnO, main peaks coincide with hexagonal structure peaks and match with JCPDS file No:36-1451. X-ray diffraction patterns of TiO_2_ nanoparticles are investigated on Joint Committee on Powder Diffraction Standards (JPDCS). Based on the results, it can be seen that TiO_2_ nanoparticles have anatase crystal structure. Scherrer equation was applied, assuming a shape factor of 0.9, so as to estimate the average crystallite size using the TiO_2_ and ZnO reflections as follows:


(4) $$D=\frac{\left(0.9\lambda \right)}{\beta \text{cos}\theta }$$

For ZnO, λ = 0.1542 nm and 2θ = 31.6 ° and B = 0.5 ° for CuKα were read from XRD pattern. For TiO_2_, λ = 0.1542 nm and 2θ = 25.2 ° and B = 0.8 ° for CuKα were read. By using Scherrer equation, average crystallite size of ZnO was found as 16.54 nm, and for TiO_2_, it was found as 10.19 nm.

**Figure 3:**
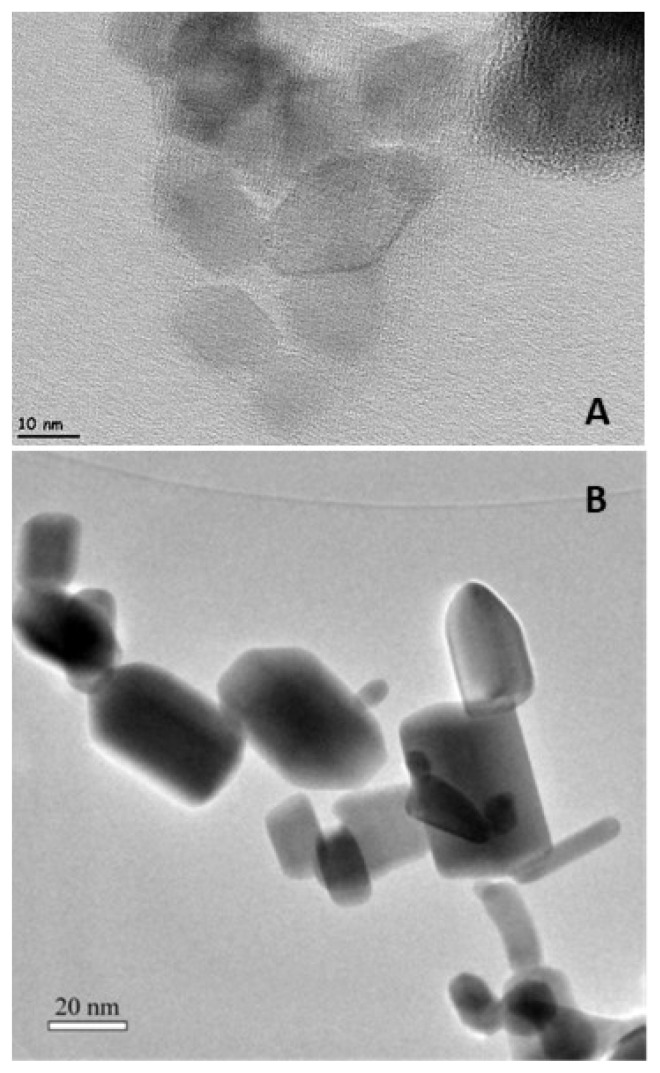
TEM images of TiO_2_ (A) and ZnO (B).

TEM images of ZnO and TiO_2_ particles are shown in Figure 3. The average particle size of ZnO nanoparticles is in the range of 30–50 nm. The size of TiO_2_, on the other hand, is between 15 and 25 nm. Besides that, it can be seen that TiO_2_ has mainly tetragonal structure. This situation is in accordance with the anatase form of TiO_2_ which has been implied in XRD results. It can be seen that ZnO has a mixture of hexagonal, tetragonal, and spherical in shape.

Surface areas of TiO_2_ and ZnO were determined. Analyses were conducted by using the same method in our previous study [18]. Surface areas of TiO_2_ and ZnO are 244.3 m^2^/g and 208.481 m^2^/g, respectively. ZnO has higher surface area than that of TiO_2_. Increase in the surface area is an important factor that elevates the catalytic activity. Along with increase in porosity, the number of active centers within the pores grows; thus, catalytic effect is also expected to increase.

**Figure 4:**
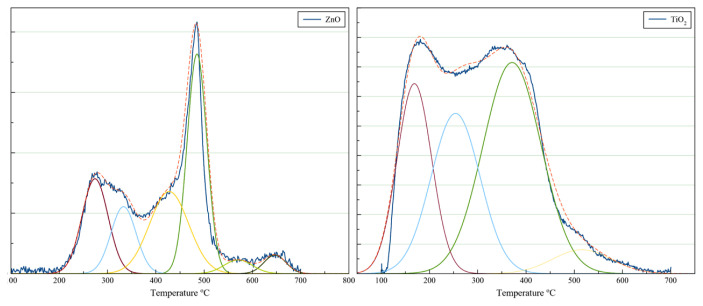
NH_3_-TPD profiles of TiO_2_ and ZnO.

Temperature programmed desorption of NH_3_ (NH_3_-TPD) was applied to characterize the acidic properties of both nanoparticles by using Micromeritics Chemisorb-2720 instrument. Thirty milligrams of powder samples were loaded in a quartz tube, placed into the furnace, and heated to 220 °C under helium flow (15 mL/min) to remove possible impurities that remain on the samples. Subsequently, the temperature was decreased to 35 °C and the system was introduced with NH_3_ (15 mL/min flow rate) for 30 min. Next, the system was purged by using helium gas with 25 mL/min flow rate for the removal of any possible NH_3_ remaining. Finally, catalysts were heated from 35 °C to 700 °C under helium flow at a heating rate of 10 °C/min and ammonia desorption was monitored by TCD. The results are shown in Figure 4.

Desorption of ammonia at high temperatures (higher than 450 °C) corresponds to strong acid sites. Desorbed ammonia between 100 and 250 °C indicates the presence of weak acidic sites. For medium acid sites, desorption temperature is in the range of 250–450 °C [19]. NH_3_-TPD profiles of both catalysts have been deconvoluted so as to investigate acidic properties. TiO_2_ has shown four peaks centered at 168, 254, 371, and 515 °C. ZnO has shown five peaks centered at 273, 333, 427, 485, 570, and 646 °C. As a result, TiO_2_ includes all types of acid sites, whereas ZnO has only medium and strong acidic sites. The acidities of TiO_2_ and ZnO were found as 0.7811 mmol/g and 0.1138 mmol/g, respectively. Therefore, TiO_2_ possesses higher amount of acidity than that of ZnO.

### 3.2 The effect of the catalyst amount

**Figure 5:**
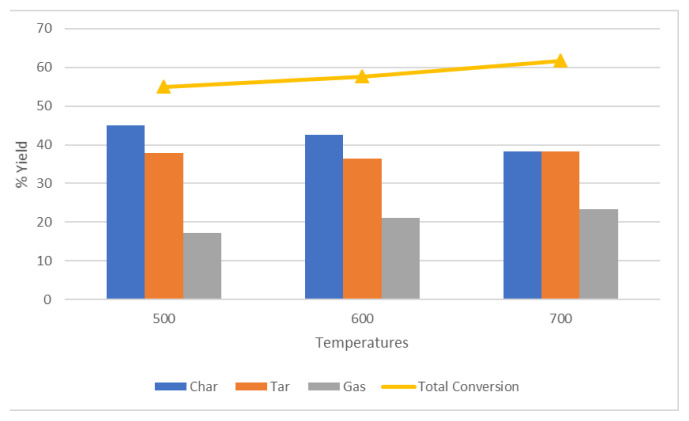
Effects of different temperatures on total conversion and product distribution in catalyst-free experiments.

Pyrolysis of cellulose was firstly conducted without catalyst. Solid, liquid, and gaseous product yields and total conversion is depicted in Figure 5. It is clear that the solid yield decreases with increasing temperature, while the gas yield increases.

Total conversion was calculated by adding up the percentages of the liquid and gaseous products. According to the results of catalyst-free experiments, highest total conversion was determined at 700 °C with total conversion yield of 61.65%.

**Figure 6:**
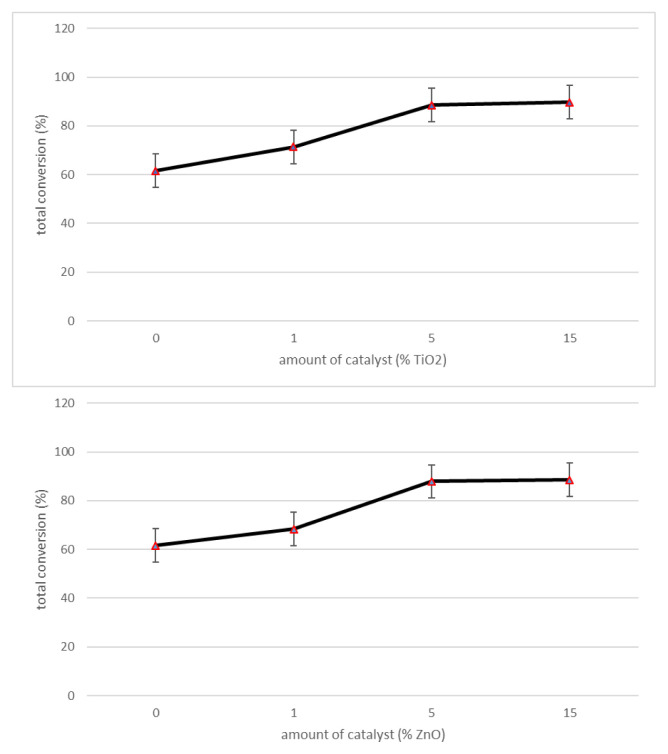
Effects of different amount of catalyst on total conversion at 700 °C.

Since the highest total conversion was determined at 700 °C (61.65%), this temperature was applied to understand the effects of catalyst amount. Yields of the experiments with different amount of TiO_2_ and ZnO nanoparticles (0%, 1%, 5%, and 15%) at 700 °C are given in Figure 6.

It can be seen from Figure 6 that total conversion increases as the TiO_2_ amount increases. There was no significant change observed when the amount of TiO_2_ and ZnO were increased above 5%. Considering the economic costs of nanosized catalysts, optimum amount of the catalysts was determined as 5%.

### 3.3. Product yields

Based on the results of catalytic and catalyst-free runs, catalytic effects on product yields are listed in Table 1. As can be seen from the experiments conducted at 500 °C, TiO_2_ increased the tar yield, while ZnO increased the yield of gaseous products noticeably. Based on the results at 600 °C, total conversion was increased on every experiment compared to the ones without catalyst. TiO_2_, most particularly, exhibited a significant increase on gaseous product yield (107%) compared to catalyst-free experiments but decreased the tar and char yields. Maximum activities of both catalysts were observed at 700 °C. At this temperature, char yield was remarkably reduced compared to catalyst-free experiments. This situation led to an increase on the yields of liquid and gaseous products. The 51.19% yield of gaseous product, in the presence of TiO_2_, was the highest gas yield obtained among all experiments.

### 3.4 Pyrolytic product distribution

In order to identify the chemical compounds in the pyrolytic products, GC-MS and micro-GC analyses were conducted. Micro-GC analyses were performed at 500, 600, and 700 °C, with and without catalysts to identify the content of gaseous product. The results are shown in Table 2. It is inferred that H_2_, CH_4_, CO_2_, CO, C_2_H_4_, C_2_H_6_, and C_3_H_8_ were the main products. All catalysts increased the hydrogen yield at 500 °C. Carbon dioxide and carbon monoxide constitute the major part of the gas composition in all experiments carried out at this temperature. A small amount of butane gas was obtained in catalyst-free experiments and in the presence of TiO_2_ catalyst. According to the results obtained at 600 °C, ZnO catalyst reduced the hydrogen yield compared to catalyst-free experiments. On the other hand, TiO_2_ increased the hydrogen yield. When this information is evaluated together with the decrease in solid yield, it can be said that the char elimination reaction (C+H_2_O ↔ CO + H_2_) can be said to be catalyzed in the presence of metal oxide [20]. As a result of increasing the pyrolysis temperature by 100 °C, acetylene gas was obtained in all catalytic and catalyst-free experiments except the ones without the usage of ZnO catalyst.

In catalytic experiments conducted at 500, 600, and 700 °C, the presence of metal oxide catalysts decreased the char yield, while they increased the tar and gas yields. This effect is attributed to specific heat capacities (SHC) and thermal conductivity coefficients (TCC) of the catalysts. The SHC for cellulose is 0.37 kcal/kg °C, for TiO_2_ it is 0.16 kcal/kg °C, and for ZnO, it is 0.11 kcal/kg °C [21]. Heat capacity is the amount of heat energy which is required to elevate the temperature of a substance per unit. In pyrolysis process, there is no significant difference between the SHC values of TiO_2_ and ZnO. Therefore, both catalysts need approximately the same heat energy to elevate their temperature. However, the difference in pyrolytic product distribution is attributed to the thermal conductivity levels. These values mean that both TiO_2_ and ZnO can easily transfer the heat to cellulose and increase its temperature quickly. On the other hand, thermal conductivity coefficients are different. For cellulose, it is 0.23 W/mK, for TiO_2_ it is 7.4 W/mK, and for ZnO it is 23.4 W/mK [22]. These values mean that if there is no metal oxide catalyst present in the pyrolysis process, transfer of the heat from reactor furnace to the cellulose is quite poor when it is compared to catalytic pyrolysis experiments [23]. Due to the great gap of TCC values between ZnO and cellulose, addition of 5% ZnO catalyst has increased the bio-oil yield because ZnO has utilized the transfer of the heat to cellulose. Additionally, the gas yield was increased when 5% TiO_2_ was used as a catalyst because of its relatively poor heat transfer since it has lower thermal conductivity coefficient than that of ZnO catalyst. As mentioned above, both catalysts have acidic nature. An increase in the tar and gas yield can be explained by acid-catalyzed cracking reactions. As a result of the cracking reactions, heavy tar turns into light tar and hydrogen [17,24]. Additionally, reactivity titanium has lower reactivity than carbon in spite of zinc. TiO2 can be reduced to metallic titanium, while carbon (char) oxidized to carbon dioxide during the pyrolysis procedure [17]. The increase in the amount of carbon dioxide supports this argument.Based on the data from GC-MS analyses, tar with high content of furanic structure was obtained and is listed in Table 3. Previous studies have shown that furanic compounds are formed as a result of dehydration of carbohydrates and the reaction can be catalyzed with acids [11, 23]. The liquid product with maximum furanic structure was obtained at 500 °C in the presence of TiO_2_ catalyst (87.98%). The amount of furanic structures in organic phase reached 87.98% as a result of the increase in furanic aldehydes and furanic alcohols, in the presence of TiO_2_ catalyst at 500 °C. This result might be attributed to the acidic properties of TiO_2_ catalyst because TiO_2_ has higher acidity and as a result, furanic structures were obtained in higher amounts at lower temperatures.

The formation of active cellulose is the first step of cellulose pyrolysis. Next, active cellulose chain is broken down to the sugar level. The first anhydrous monosaccharide formed is levoglucosan (LGA). LGA then undergoes isomerization and dehydration reactions and as a result, LGA can be transformed into other types of anhydrosugars. According to GC-MS results, LGA was completely eliminated thanks to the acidic surface of the obtained catalysts [25]. Quantities of furanic aldehydes and furanic alcohols are depicted in Table 3. Catalytic pyrolysis of HMF was investigated by using zeolites and results have shown that aromatic hydrocarbons can be obtained from HMF [26]. In the presence of TiO_2_ catalyst, HMF was eliminated and thought to be converted into aromatic hydrocarbons and it was supported by the GC-MS results. TiO_2_ catalyzed the degradation of HMF and increased the yield of aromatic hydrocarbons. It has been reported that acidic catalysts enhance the dehydration and decarbonylation reactions and this result coincides with our study because 2-furanmethanol yield was increased as the carbon monoxide yield increased [27].

Liquid products that contain high amount of furanic structures were obtained at low temperatures. In all experiments conducted, amount of furanic aldehydes and furanic alcohols were found to be higher than other structures. In the presence of nanometal oxide catalysts, amounts of these products were increased compared to catalyst-free experiments.

## 4. Conclusion

In catalyst-free cellulose pyrolysis, the temperature with the highest liquid and gas yields was determined at 700 °C. For this reason, effect of catalyst amount was put into practice at this temperature using 1%, 5%, and 15% catalysts. The optimum yield was achieved by using 5% catalyst. In the presence of TiO_2_ at 700 °C, maximum gas yield was achieved. GC-MS results showed that the nanometal oxide catalysts reduced the product distribution. The presence of metal oxide catalysts decreased the char yield but increased the tar and gas yields at 500, 600, and 700 °C. Obtained liquid phase contains high amount of furan derivatives with low molecular weight. Sulfated metal oxides for cellulose pyrolysis aimed to achieve low molecular weight furan compounds [11]. In this study, nanosized metal oxides were used without any extra treatment to provide an acidic structure whose effectiveness in pyrolysis is already known. As a result, we obtained high rates of furanic compounds with low molecular weight.

## Figures and Tables

**Table 1:** Effects of 5% TiO2 and 5% ZnO catalysts at different temperatures on product yields.

	500 °C			600 °C			700 °C		

	No Cat.	5% TiO_2_	5% ZnO	No Cat.	5% TiO_2_	5% ZnO	No Cat.	5% TiO_2_	5% ZnO

Char	44.97 ± 2.42	33.89 ± 3.10	30.84 ± 1.71	42.48 ± 3.86	30.84 ± 2.14	24.23 ± 1.67	38.36 ± 4.08	11.41 ± 1.48	12.08 ± 1.61
Tar	37.92 ± 2.87	41.95 ± 2.77	36.95 ± 3.11	36.34 ± 3.11	25.37 ± 1.98	39.77 ± 3.05	38.29 ± 3.68	36.68 ± 2.39	41.74 ± 3.91
Gas	17.11 ± 1.94	24.16 ± 1.93	32.21 ± 2.58	21.17 ± 2.97	43.79 ± 2.16	36.01 ± 2.22	23.36 ± 2.98	51.91 ± 4.73	46.17 ± 4.47

**Table 2:** Obtained gaseous products as mg gas/g catalyst.

	No Cat. 500 °C	No Cat. 600 °C	No Cat. 700 °C
**CO** **_2_**	97.77	115.10	122.53
**CO**	61.11	75.98	88.68
**H** **_2_**	0.69	3.12	4.95
**CH** **_4_**	6.39	10.45	11.70
**H** **_2_** **O**	0.32	0.44	0.49
**C** **_2_** **H** **_6_**	1.90	2.80	2.36
**C** **_2_** **H** **_4_**	1.09	1.80	1.73
**C** **_3_** **H** **_8_**	1.10	1.76	1.07
**C** **_2_** **H** **_2_**	0.00	0.01	0.002
**C** **_4_** **H** **_10_**	0.780	0.278	0.054
	**5% TiO_2_ 500** °**C**	**5% TiO_2_ 600** °**C**	**5% TiO_2_ 700** °**C**
**CO** **_2_**	141.15	231.11	273.20
**CO**	84.32	163.96	196.31
**H** **_2_**	1.34	8.57	10.14
**CH** **_4_**	7.40	16.71	25.97
**H** **_2_** **O**	1.8	0.96	0.94
**C** **_2_** **H** **_6_**	2.38	4.86	4.52
**C** **_2_** **H** **_4_**	1.34	3.55	3.37
**C** **_3_** **H** **_8_**	1.61	4.81	3.14
**C** **_2_** **H** **_2_**	0.00	0.03	0.01
**C** **_4_** **H** **_10_**	0.989	3.370	1.517
	**5% ZnO 500** °**C**	**5% ZnO 600** °**C**	**5% ZnO 700** °**C**
**CO** **_2_**	220.7	202.25	228.36
**CO**	85.28	131.82	186.81
**H** **_2_**	3.16	4.33	9.49
**CH** **_4_**	6.53	12.44	26.47
**H** **_2_** **O**	0.76	1.15	2.13
**C** **_2_** **H** **_6_**	2.46	3.21	3.43
**C** **_2_** **H** **_4_**	1.52	2.32	2.80
**C** **_3_** **H** **_8_**	1.68	2.55	2.25
**C** **_2_** **H** **_2_**	0.00	0.00	0.00
**C** **_4_** **H** **_10_**	0.00	0.00	0.00

**Table 3:** % peak areas of furanic structures in bio-oil.

	500 °C			600 °C			700 °C		

	No Cat.	5% TiO_2_	5% ZnO	No Cat.	5% TiO_2_	5% ZnO	No Cat.	5% TiO_2_	5% ZnO

**Alkyls/Aryls**	2.75	5.81	5.28	1.09	4.16	1.96	2.05	3.65	3.14
**Alcohols**	10.92	28.18	11.11	-	30.12	11.34	-	19.38	13.59
**Aldehydes**	21.50	45.57	55.08	47.48	37.20	64.06	46.19	39.25	55.74
**Ketones**	2.69	4.06	2.27	5.03	5.77	3.17	4.60	4.27	2.68
**Esters**	1.71	4.36	3.54	1.90	4.07	3.18	1.47	2.96	4.00
**Furanic acids**	-	-	0.88	-	-	-	-	2.29	1.17
**Other furanic structures**	-	-	3.08	-	-	-	-	-	-
